# Educational interventions to improve people’s understanding of key concepts in assessing the effects of health interventions: a systematic review protocol

**DOI:** 10.1186/s13643-016-0213-9

**Published:** 2016-02-25

**Authors:** Leila Cusack, Chris B. Del Mar, Iain Chalmers, Tammy C. Hoffmann

**Affiliations:** Faculty of Health Sciences and Medicine, Centre for Research in Evidence-Based Practice (CREBP), Bond University, Robina, QLD 4229 Australia; James Lind Initiative, Oxford, UK

**Keywords:** Consumer, Education, Health information, Health literacy, Critical health literacy, Critical appraisal

## Abstract

**Background:**

Health information has become readily accessible through mass media, and people are playing a more active and autonomous role in their health. Much of the health information that was previously only available to health professionals is now directly accessible to the public. Consequently, people often navigate vast amounts of health information on their own, typically with little knowledge about how to evaluate it or the need to do so.

Health information remains essentially unregulated, and widespread problems and concerns with the quality of health information have been noted. In addition to the variable quality of health information, inconsistent and/or inappropriate use of related terminology (e.g. ‘evidence-based’ and ‘clinically proven’) can be confusing to the public, who are ill-prepared to critically examine claims.

The general public are not trained in the fundamentals of health research and do not typically possess the knowledge and skills to evaluate the accuracy and completeness of information about health interventions. Without this, the public are vulnerable to acting on inaccurate or incomplete health information and making ill-informed health decisions. With this review, we intend to identify and assess educational interventions which have been designed to improve people’s ability to understand key concepts relevant to evaluating claims about the effects of health interventions.

**Methods/design:**

This systematic review of the literature will use a search strategy that has been developed in conjunction with a Health Sciences Librarian who has expertise in systematic review searching to identify relevant studies. Databases to be searched include the following: the Cochrane Central Register of Controlled Trials, MEDLINE, EMBASE, CINAHL, and ERIC. Attempts to identify unpublished studies and ongoing trials will also be made. Two review authors will independently screen search results and assess studies for eligibility. Studies which aim to improve participants’ understanding of the key concepts relevant to evaluating the effects (or the interpretation of results) of health interventions will be included. Randomised trials, non-randomised trials, controlled before and after studies, controlled studies with only post-test measures, and interrupted time series studies will be eligible for inclusion. We will contact study authors to clarify any missing details/data. Due to the nature of the systematic review question and the expectation of heterogeneity in study design, interventions, and outcomes, we intend to take a narrative approach to data synthesis.

**Systematic review registration:**

PROSPEROCRD42016033103

## Background

Health information, and misinformation, is readily accessible to people, particularly through mass media and the Internet [1–6]. Due to the ease with which large amounts of health information can now be accessed, people are playing a more active and autonomous role in their health [7]. The health information that people access can affect their health decisions and behaviours—for example, from how they maintain their health and cope with a chronic condition to what decisions are made about how to treat an illness or whether to consult a health professional [8, 9].

As well as websites and traditional information sources such as magazines, radio, and television, health information is also available on social media such as Facebook [10], YouTube health channels, and Twitter [11]. Regardless of the type of media, health information remains essentially unregulated and problems and concerns with the quality of health information have been noted [2, 12, 13].

Traditionally, people relied on health professionals as intermediaries between themselves and health information. However, health information that was previously only available to health professionals is now directly accessible to the public. Consequently, people often navigate vast amounts of information on their own, typically with little knowledge about how to evaluate it or the need to do so [14].

Health interventions are one of the most commonly researched health topics [8, 9], yet the quality of health information is variable [5]. Some interventions are promoted using phrases such as ‘evidence-based’ and ‘clinically proven’. Phrases such as these are intended to convince people of an intervention’s effectiveness, and when the claims are not warranted, their use can be misleading. Some of the other complexities that can influence people’s decision-making about interventions are that some people tend to rely on anecdotes rather than information derived from research [15, 16], some overrate the trustworthiness of the health information they find and most overestimate the benefits and underestimate the harms of interventions [17]. Belief in false or unsubstantiated claims about interventions may result in people receiving inappropriate interventions that are at best ineffective and at worst harmful, as well as wasting health care resources. Conversely, not believing claims that are based on reliable research evidence about interventions can also cause harm, such as through inappropriate treatments or delays in seeking appropriate health care [18–21] or choosing ineffective treatments over effective ones [22].

Health information may be misleading, misinterpreted, or leave people confused [2, 23].

Consumers require skills in assessing the quality of both general health information and information about health interventions and their effectiveness. Without specific education about key concepts relevant to evaluating the effects of health interventions and how to interpret research results, people are, irrespective of their level of education, vulnerable to believing health claims and may make health decisions based upon information that is inaccurate, incomplete, or even harmful [19, 22]. Considering its nature and extensive reach, regulation of health information on the Internet, or other sources, is impossible. However, providing people with knowledge about key concepts in evaluating information about health interventions may assist them to evaluate the accuracy of intervention claims and to make informed decisions. Unlike most health professionals, the general public are typically not trained to evaluate the accuracy and completeness of information about health interventions [14].

Most of the existing research in the area of helping people to understand health information has focused on the traditional skills associated with health literacy, such as reading, numeracy, and oral literacy. Limitations in these skills can impact upon people’s ability to navigate the health system and are associated with poorer outcomes and decreased uptake of provided health services [24]. Previous systematic reviews have examined the effectiveness of related interventions such as teaching online health literacy to the general public [25] and critical appraisal skills to health professionals [26], and a review to assess the effects of educational interventions on critical appraisal abilities in school students is currently underway [27]. Yet, to our knowledge, there is no systematic review of studies of educational interventions designed to improve critical appraisal abilities in the general public. In this review, we will identify, appraise, and synthesise studies that have evaluated educational interventions which cover one or more key concepts in assessing the effects of health intervention concepts in the critical appraisal of health intervention claims.

### Objectives

The objective of this systematic review is to assess the effectiveness of educational interventions designed to improve people’s understanding of key concepts (described below) when evaluating claims about the effects of health interventions.

## Methods

### Eligibility criteria

#### Study designs

Randomised trials, non-randomised trials, controlled before and after studies, controlled studies with only post-test measures, and interrupted time series studies will be eligible for inclusion. As this type of educational intervention is relatively new, we anticipate that there will be few randomised trials, but there may be other comparative studies.

#### Participants

Studies of educational interventions directed at the following populations will be eligible for inclusion: patients, members of the general public, journalists, and school or university students who are not undertaking health studies. There is no minimum or maximum limit on the age of participants. Studies in which all participants are either tertiary health students and/or health professionals will be excluded from the review, as an educational intervention provided to them on this topic is most likely designed to assist them to perform decision-making with their patients, rather than decision-making regarding their own health.

#### Intervention

Any educational intervention which aims to help the participants understand one or more of the key concepts that are relevant to evaluating the effects of health interventions and/or the interpretation of research results will be eligible. The educational intervention might be focused and only address one concept (such as the rationale for randomisation) or it may address multiple concepts. A list of the key concepts necessary to assess claims of the effects of a treatment will inform the concepts eligible for this review. This concept list has been developed by Austvoll-Dahlgren and colleagues [24] based on the contents of *Testing Treatments* [28], a book written for the public, and consultations with a wide range of commentators. Examples of concepts that may be covered include the following: how and why health research is performed, different kinds of study types and ‘evidence’, what makes a study valid and reliable, concepts such as the role of chance, the role of randomised trials and systematic reviews, and how to interpret study results. More specific concepts could include the following: PICO questions, randomisation, concealed allocation, equipoise, comparison groups, the role of blinding in outcome assessment, placebos, understanding outcomes (e.g. absolute vs relative risk), and bias and conflicts of interest in research.

This information may either be the focus of the intervention or used as an example/scenario to illustrate broader scientific knowledge (e.g. causation vs association). The examples/scenarios used must be within the context of health information or claims, health conditions, the human body, and/or conventional, complementary, or alternative medical treatments to be eligible. There will be no restriction on other characteristics of the educational interventions, such as the mode of delivery (e.g. face-to-face or remotely, by the internet or written materials), whether the intervention was delivered in a group or one-to-one, the intensity and duration of the intervention (such as one session or multiple sessions), and whether it is provided using didactic or interactive methods.

#### Comparators

There will be no restriction on the comparator used in eligible studies. For example, this may include no intervention, an ‘attention control’ intervention, or an educational intervention that covers different topics.

#### Outcomes

Primary outcomes will include any measures that evaluate knowledge or understanding of concepts relevant to evaluating the effects of, or claims about, health interventions. Secondary outcomes will be any measures of application of the knowledge taught, through demonstrations of relevant skills, behaviours, and attitudes, as well as satisfaction with the intervention.

#### Timing

There will be no specific eligibility criteria for inclusion based on when post-intervention outcomes are measured.

#### Setting

There will be no restrictions by type of setting.

### Language

There will be no language restrictions in our search strategy. We will attempt to translate potentially eligible non-English articles via Google Translate or by a native speaker of the language of the article. In the event that an article is eligible but unable to be satisfactorily translated, we will present the title and author details in a supplementary appendix.

### Information sources

#### Electronic databases

Search strategies will be developed using appropriate Medical Subject Headings (MeSH) and text words. We will search the following electronic databases:

- The Cochrane Central Register of Controlled Trials (CENTRAL The Cochrane Library, Issue 10 of 12, October 2015)
- Cochrane Consumers and Communication Review Group Specialised Register (accessed November 2015)
- MEDLINE (OvidSP) (1946 to November 2015)
- Embase.com (1966 to November 2015)
- CINAHL (1982 to November 2015)
- Web of Science Core Collection (1900 to November 2015)
- ERIC (1990 to November 2015)
- Searches of the International Clinical Trials Register (ICTRP) Search Portal and ClinicalTrials.gov will be conducted to identify trials that are ongoing (present).

#### Other resources

Sources of grey literature that will be searched are as follows:

Relevant organisations, such as the following:

- Health Literacy Europe (healthliteracyeurope.net)
- Health Literacy UK (healthliteracy.org.uk)
- Health Literacy Research Network (healthliteracy.net.au)

Relevant theses and dissertations, via the following:

- National Library of Australia’s Trove service (http://trove.nla.gov.au/)
- Networked Digital Library of Theses and Dissertations (http://www.ndltd.org/)
- ProQuest Digital Dissertations and Theses (http://www.proquest.com/products-services/pqdt.html)

We will perform forward and backward citation searching of eligible studies. We will attempt to locate unpublished studies by contacting published authors in the field and asking if they are aware of ongoing and unpublished studies.

### Search strategy

No date or language limits will be imposed on the search.

The specific search strategies were created by a Health Sciences Librarian with expertise in systematic review searching. The MEDLINE (OvidSP) strategy (shown in the Appendix) will be adapted for the other databases

### Study records

#### Data management

All search results will be merged into the reference management software EndNote, and duplicate records of the same report will be removed using the Centre for Research in Evidence-Based Practice Systematic Review Assistant ‘deduplication tool’ [29].

#### Selection process

Two researchers will independently assess the eligibility of studies by screening titles and abstracts for potential inclusion according to predefined selection criteria. Studies judged to be potentially relevant will be retrieved in full text for further analysis. Any disagreements in judgement will be resolved by discussion to reach a consensus or, if this is not possible, with a third review author until consensus is reached. If further information about the study is required in order to make a decision about its eligibility, an attempt will be made to contact the study corresponding author(s). If a response is not received after three reminders are sent and/or after attempting to contact another author of the paper with no response, the study will be excluded. We will also provide citation details and any available information about ongoing studies.

#### Data collection process

Two review authors (LC and TH) will extract data independently from included studies. Where necessary, study authors will be contacted to provide additional information about the study method or results. All extracted data will be entered into Review Manager (RevMan 2012) by one review author (LC) and will be checked independently by a second review author (TH) for accuracy against the data extraction sheets. Any discrepancies will be resolved by discussion, or through consultation with a third author (CDM), until consensus is reached.

### Data items

Where possible, extracted data will include study details, demographic, methodology, intervention and comparison details, and all relevant outcomes and results:

- Study details: aim, design, funding sources
- Participants: recruitment, inclusion/exclusion criteria, geographic location, total numbers (including those excluded, withdrawn, or lost to follow-up), age, gender, ethnicity, health problems, educational attainment, and socioeconomic status.
- Intervention: theoretical basis, aim, content, method of delivery and other format details, where intervention was provided, details of materials used in the intervention and if/where they can be accessed, duration and schedule of intervention, details of those who provided the intervention, and intervention fidelity (including any monitoring of this throughout the study).
- Control: details of the control condition and, if an ‘active comparator’, details of it as per the elements recorded for the experimental intervention.
- Outcomes: primary and secondary measures, methods used to collect outcome data, validity and reliability of outcome measures, methods of follow-up for non-respondents, and timing of outcome assessment
- Results: data (descriptive statistics and summary results) for each eligible outcome measure.

### Outcomes and prioritisation

Primary outcomes will demonstrate an understanding of the knowledge taught and will include any measures that evaluate knowledge or understanding of concepts relevant to evaluating the effects of, or claims about, health interventions. For example, this may include the following:

- Understanding the need for critical appraisal
- Recognising the need to consider all relevant and reliable evidence
- Understanding concepts that underpin health intervention research (such as randomisation and causation)
- Recognising the need for systematic reviews, preferably of randomised trials
- Understanding the role of chance and understanding the results of trials and systematic reviews and
- Judging whether a trial or systematic review is relevant

Secondary outcomes will demonstrate an application of the knowledge taught and will include any measures of the following:

- Confidence in applying any of the knowledge concepts, for example, this might be demonstrated through rating the quality of health information and intervention claims in academic or non-academic sources (such as media stories, advertisements, and websites)
- Behaviours (actual or hypothetical) related to making decisions about health interventions, such as whether to have a particular intervention
- Attitudes towards the need for critically appraising health information and intervention claims and
- Attitudes towards, satisfaction with, or completion of the intervention

Due to the nature of this review, we expect variety in the types of outcome measures used. For example, the knowledge outcome may be assessed through multiple choice questions or written responses to scenarios. The timing of when the measures are administered is also likely to vary between studies. For example, some outcomes may be assessed pre- and post-intervention and others at only post-intervention. These variations are likely to limit pooling of data and restrict synthesis to a narrative summary.

### Risk of bias in included studies

To assess and report on the possible methodological risk of bias of included studies, ACROBAT-NRSI (A Cochrane Risk Of Bias Assessment Tool: for Non-Randomized Studies of Interventions) guidelines [30] will be used. These guidelines are a modified version of the Cochrane risk of bias tool [31] and are specifically designed for non-randomised studies of interventions. The topics will include the following: sequence generation, allocation concealment, blinding, incomplete outcome data (e.g. dropouts and withdrawals), selective outcome reporting, and other areas of bias. For each domain in the tool, the procedures undertaken for each study will be described. Each study will be rated as ‘high risk’ or ‘low risk’ of bias based on a judgement of the gathered information. If there is insufficient detail reported in the study, the risk of bias will be classified as ‘unclear’ and the original study investigators will be contacted for more information. These judgements will be made independently by two review authors (LC and TH) based on the criteria for judging the risk of bias [32]. Disagreements will be resolved by discussion or, if necessary, through arbitration with a third author.

### Data synthesis

Where studies are sufficiently homogeneous in terms of design and comparator, we will conduct meta-analyses using a random-effects model. However, due to the nature of this study (with expected substantial heterogeneity detected across included studies, in relation to study design, intervention design and type, and outcomes), it is anticipated that the results will not be conducive for a meta-analysis. With the expectation of heterogeneic results, we intend to tabulate results and present an appropriately clustered descriptive synthesis of the eligible studies.

In studies which compare more than one intervention, each will be compared separately to no intervention/control and also with one another.

#### Dealing with missing data

Attempts will be made to contact study authors to obtain any missing data (e.g. participant, intervention, or outcome details). Analyses will be conducted (where possible) on an intention-to-treat basis; alternatively, data will be analysed as reported. Losses to follow-up will be reported and assessed as a potential source of bias.

#### Assessment of heterogeneity

Where studies are considered appropriately similar (based upon populations, interventions, and outcomes) to allow pooling of data using meta-analysis, the degree of heterogeneity will be assessed by visual inspection of forest plots and also by the Chi^2^ test for heterogeneity. Heterogeneity will be quantified using the *I*^2^ statistic. The *I*^2^ value (with 50 % or more representing substantial levels of heterogeneity) will be interpreted in light of the size and direction of effects and the strength of the evidence for heterogeneity, based on the *P* value from the Chi^2^ test [33].

#### Sensitivity analysis

If there is a sufficient number of studies, we will conduct a sensitivity analysis as appropriate (for example, results from randomised trials vs results from other study designs).

### Assessment of meta-bias(es)

Reporting bias will be assessed qualitatively based upon the characteristics of the included studies (e.g. if only small studies that indicate positive findings are identified for inclusion) and also based upon the information that we obtain from experts and authors which suggests that relevant studies exist yet remain unpublished. If a sufficient number studies (at least 10) are identified for inclusion in the review, we will construct a funnel plot to examine small study effects, which may demonstrate the presence of publication bias. Funnel plot asymmetry will be tested formally, with the selection of test based on the advice in Higgins and Green [33], remaining mindful of the fact that there may be several reasons for funnel plot asymmetry when interpreting the results.

### Strength of evidence: confidence in cumulative estimate

Where possible, the quality of the evidence will be assessed and reported using the Grading of Recommendations Assessment, Development and Evaluation (GRADE) working group methodology. The quality of evidence will be assessed across the domains of risk of bias, consistency, directness, precision, and publication bias. Quality will be considered either as high (further research is very unlikely to change our confidence in the estimate of effect), moderate (further is likely to have an important impact on our confidence in the estimate of effect and may change the estimate), low (further research is very likely to have an important impact on our confidence in the estimate of effect and is likely to change the estimate), or very low (very uncertain about the estimate of effect). Two authors (LC and TH) will independently assess the quality of the evidence as implemented and described in the GRADEprofiler (GRADEpro) software.

## Discussion

As people seek health information more actively and independently, knowledge about the key concepts that should be considered when evaluating claims about the effects of health interventions may assist people to make appropriately informed decisions. An understanding of the effects of existing interventions that aim to provide this type of education will be useful in informing the development of future educational interventions. Additionally, if few eligible studies are identified, this review will draw attention to an important area that requires further research effort.

### Presenting and reporting of results

This protocol follows the Preferred Reporting Items for Systematic Review and Meta-Analysis Protocols (PRISMA-P) 2015 Statement [34]. We will present the results of this review according to the Preferred Reporting Items for Systematic Reviews and Meta-Analyses (PRISMA) guidelines, using a flow diagram to report the identification and selection of studies. The relevant outcomes and characteristics of each study will be reported in summary tables. Where statistical pooling is not possible, the findings will be alternatively presented in narrative form including tables and figures to aid in data presentation where appropriate.

### Interpretation of findings

The results of the review will be discussed in the context of the quality of the evidence, the limitations of the review, and the strengths of findings as well as their implications for current practice and future research directions.

## Appendix

Medline search (Ovid interface)

exp Persons/OR exp Patients/OR (People OR Patient OR Patients OR People OR Schoolchildren OR Student OR Students OR Public OR Consumer OR Consumers OR Participant OR Participants).tw.

AND

exp Health Education/OR exp Teaching/OR Education.tw. OR Educational.tw. OR Teaching.tw. OR Taught.tw. OR Train.tw. OR Trained.tw. OR Training.tw. OR Workshop.tw. OR Workshops.tw.

AND

((exp Evidence-Based practice/OR exp Information literacy/) AND (Understand OR Understanding OR Competency OR Competencies OR Appraise OR Appraisal OR Skill OR Skills OR Evaluate OR Evaluation OR Knowledge).tw.

OR

(Evidence based OR Evidence-based OR Critical OR Critically OR Health risk OR Health risks OR Randomisation OR Randomization OR Control group OR Comparison group OR Health information OR Health literacy OR Health information OR Healthcare information OR Medical information OR Health advice OR Healthcare advice OR Medical advice OR Health research OR Healthcare research or Medical research OR Statistics OR Controlled trial OR Controlled trial OR Clinical trial OR Clinical trials OR RCT OR RCTs OR Systematic review OR Systematic reviews).tw. adj2 (Understand OR Understanding OR Competency OR Competencies OR Appraise OR Appraisal OR Skill OR Skills OR Knowledge OR Evaluate OR Evaluating OR Evaluation OR Assess OR Assessing OR Think OR Thinking).tw.)

AND

(Randomized Controlled Trial or Controlled Clinical Trial or Multicenter Study).pt. or exp Epidemiologic Studies/or Program Evaluation/or Pilot Projects/or (Randomised or Randomized or Randomise or Randomize or Randomly or Random allocation).tw. OR ((Group OR Groups) adj2 (random* or between or control or intervention)).ab. OR Controlled trial.tw. or (intervention* or controlled or control group or compare or comparison* or compared or ((prospectiv* or crossover) adj2 (study or studies or design)) or (before adj2 after) or (pre adj2 post) or pretest or pre test or posttest or post test or quasiexperiment* or time series or quasi experiment* or evaluat* OR effectiveness or impact or time series or time point? or repeated measur*).tw. or Observational.tw. or Observe.tw. or Observation.tw. or Observing.tw. or Survey.tw. or Surveys.tw. or Surveyed.tw. OR Interviews.tw. OR Comparison.tw.

NOT

exp Health Personnel/OR exp Education, Professional/OR exp Students, Health Occupations/

OR

Review.pt. OR Meta Analysis.pt. OR News.pt. OR Comment.pt. OR Editorial.pt. OR Letter.pt. OR cochrane database of systematic reviews.jn. OR comment on.cm. OR (systematic review or literature review).ti.

NOT

exp Animals/not (exp Animals/and Humans/).

## Abbreviations

ACROBAT-NRSI: A Cochrane Risk Of Bias Assessment Tool: for Non-Randomized Studies of Interventions
GRADE: Grading of Recommendations Assessment, Development and Evaluation
GRADEpro: GRADEprofiler
ICTRP: International Clinical Trials Register
MeSH: Medical Subject Headings
PRISMA: Preferred Reporting Items for Systematic Reviews and Meta-Analyses
PRISMA-P: Preferred Reporting Items for Systematic Review and Meta-Analysis Protocols
RevMan: Review Manager
